# Hemorrhage

**Published:** 1856-06

**Authors:** 


### IOWA MEDICAL JOURNAL.

VOL. III. KEOKUK, IOWA, MAY AND JUNE, 1856. NO. 3.

### ORIGINAL COMMUNICATIONS.

### HEMORRHAGE FROM MUCUS SURFACES ATTENDING THE COLD STAGE OF INTERMITTENTS.

The record is already so replete with the history and theories respecting intermittent, and other forms of malarious diseases, that to attempt to offer anything new in the way of either, is to hazard one's reputation as a well informed man in medical science. It may well be, that the history of intermittent fever contains such facts as those which I am about to offer, but if so, I have not been so fortunate as to have read of them, or having done so, have to blame a poor memory for having forgotten the fact and, per consquence, shall in the present article render myself obnoxious to the very general complaint against contributors to medical journals, that they mistake, for new, old and well known facts and for those of general moment, those which are of particular interest to themselves only. Nor can I claim, in extenuation of the error, that the complaint is ill-founded, since a perusal of the very numerous journals of the day, filled as they are, with good, indifferent, and worthless communications is suffi cient to establish its justice. The evil is increasing, too, with the inordinate multiplication of medical periodicals,; and it is but just what might be expected. They are not "paying institutions" in a pecuniary sense, and editors, with very few exceptions, intelligent and successful practitioners, cannot afford to devote all their time to conducting them. Contributions are of all qualities, but there is so much space to be filled with something, and such as they are, in they go, to make up the "original communication" department, without which no journal may make its appearance with the least prospect of a favorable reception. Subscribers and patrons, read for a time, then "look over" and finally, only "take the journals."

The custom of "reporting cases" for publication, has been and still is, a source of untold benefit to the profession, but it is, like all other good things, capable of abuse, and, when injudiciously resorted to, is productive of indirect, but positive harm. One's "case book" may contain very many, and, indeed, nothing else than cases of interest, to the maker of it, but if he were to publish them all, those would be rare instances when all would be read.

But this is not the object for which I sat down, and if anybody has been foolish enough to read the above commencement to a homily I never intended, I humbly entreat pardon for the infliction which I have been incontinently betrayed into imposing upon them. In the meanwhile let me proceed with the work before me

The summer and autumn of 1855, were, in the State of Michigan, accompanied with remarkably abundant and oft-repeated rains. To such an extent was this the case, that crops of various kinds were materially injured thereby, particularly upon low and clay lands. Malarious diseases infected the *uplands* in an unusual degree, and localities ordinarily exempt from them were particularly obnoxious to them. The lowlands where they usually prevail, during the autumnal months, were almost exempt, and in such sections of the country it was termed, and, indeed, was "a very healthy season." Such was the fact in the place where I was at the time located, and in that vicinity, which was all low land, thickly interspersed with swamps and marshes, and where, from its first settlement, this class of diseases was exceedingly prevalent. Nor was there any other class of diseases prevailing, except some few cases of variola scattered here and there among emigrants from Europe—and these cases were all of a mild character.

On the 23d of September I was called to see K. de R. and his sister, who, as the messenger informed me, had been afflicted for three days each, with a quotidian ague. They were, he said, both of them about the house, but were expecting the return of the paroxysm at about the same time, which would be in a couple of hours. Having other and previous engagements to fulfil, I did not obey the summons for some two hours, and while on the way, for that object, I met the messenger, riding in haste to urge me to lose no time in reaching the residence of the patient's, as K. de .R had been taken subsequent to the commencement of the chill, and but a few minutes since, with large evacuations of blood from the stomach and bowels; that blood was also escaping from the nose and mouth, and that he was voiding bloody urine. Upon inquiry, I learned that he and the sister had each, the evening before, by advice of a neighbor, taken a tablespoon heaped full of rhubarb, and that it had produced severe catharsis, the stools being, as he expressed it, nothing but water.

Upon my arrival, K. had just left the stool, upon examining which, I found about one and a half pints of dark, partially coagulable blood. Hæmatemesis occurred soon after—the appearance of the blood being of the same character as that passed at stool. Blood was oozing slowly from the nose and from the gums. The patient complained of a slight pain in the abdomen; was unable to bear moving or to sustain an upright position without extreme faintness. The countenance was sunken, anxious and haggard—pulse almost imperceptible at the wrist and ankles. Resperation labored and sighing, and the surface bathed in cold sweat. Administered diffusible stimulants, Quinine and styptics internally, and applied dry heat and frictions with mustard, externally. The hemorrhage became somewhat less and the pulse came up for a few minutes, but all was of no avail. He died four hours after the commencement of the discharge.

The amount of blood lost was large, but I had no means of ascertaining it with exactness, as each evacuation had been thrown hastily out after it was made, nor had there been any distinct account of the number of discharges previous to my arrival, I was informed, there had been quite a number and, his father-in-law, a very intelligent man, remarked, they had each been quite as large and some even larger than the first seen by myself and described above. The hæmatemesis had also been more copious before than after my arrival.

The sister I had found, upon my arrival, suffering from a severe chill, accompanied with pain in the abdomen, and repeated retchings. In the greater extremity of the brother I paid her little attention until I was informed by her nurse, about half an hour later, that she too had just vomited blood freely, that she was just upon the stool and she was fearful her attack was to prove identical with the brother's. She was correct in her surmise. The evacuation consisted of from eighteen to twenty ounces of the same dark blood. There was however no hemorrhage from the nasal nor buccal mucus membrane nor yet from the bladder. As in the other case, I administered styptics, diffusible stimulants and Quinine (for the pulse became exceedingly feeble) happily, checking and finally, entirely arresting the discharge and procuring proper reaction. By the ordinary treatment for intermittent afterwards she speedily recovered her health.

The following day, I was called into another neighborhood, some four miles distant to another case, (that of a man) identical to all appearance, with that of K. de R.; the hemorrhage coming on in the same manner during the cold stage and, as in his case, the discharge was from all the mucus surfaces save that of the lungs. Recovered.

The fourth case was that of a female, who had been in feeble health during the latter part of her journey, and since her arrival in this country from Europe, some six weeks previous. In this case the hemorrhage commenced precisely as in those already described, but, unike those, did not cease when reaction came on, and furthermore, in a day or two after the attack, blood was effused under the cuticle, the surface being completely mottled from head to foot with dark purple spots from this cause. Blood continued to flow slowly from the mouth and nose, at irregular intervals, for several days. She recovered under the influence of Quinine and chalybeates.

After these, there occurred eleven other cases, one of which resulted fatally. It was seen only when, as in the first one described, it was too nearly gone to be within the reach of remedial measures. The remainder were speedily recovered under the administration of Quina in full doses, together with such other remedies for the arrest of hemorrhage as are ordinarily resorted to. All the cases occurred within twelve days time from the first one attacked. After subduing the intermittent I prescribed to several of them the following, which for a number of years I have been accustomed to prescribe to prevent recurrence of the disease in those inveterate cases where it returns again and again at stated periods:

| | | |
|---|---|---|
| ℞. | Strychniæ | gr ij |
| | Acid: Sulph: Aromat | f ʒiij |
| | Spts Lavandulæ co | f ℥iijxʒv |
| | M. ft. Solutio | |

Signa. A teaspoonful thrice daily, to an adult. This may have been a useless precaution, but the disease was so fearfully formidable that I was willing, if possible, to make assurance doubly sure.

Of the fifteen cases three were females. All were, and had been throughout summer, in robust health up to the time when attacked with the ague, with the single exception of the emigrant woman named, nor had any one of them had more than three paroxysms before the one in which the hemorrhage occurred. All the males were engaged in agricultural or mechanical pursuits, while the females were wives and daughters of farmers. They were subjected to no special morbid or depressing influence more than their neighbors. They were scattered through several neighborhoods, distant some miles from each other. Save the emigrant woman no one had yet arrived at middle age. As before remarked, the season was remarkably healthy.

In the first two cases, I supposed the rhubarb to have exerted the chief influence in producing the hæmorrhage, or rather that the hypercatharsis had predisposed the alimentary mucus membrane to extravasion, and that when the usual conjestive condition of the internal organs occurred during the cold stage of ague, hemorrhage from the mucus surface already weakened by the previous discharges, resulted. This might account for the hemorrhage from the bowels, but not so well for that from other mucus surfaces. When, however, I found this peculiarity in the disease manifesting itself in other instances where no such cause was present to account for it, and that too with equal violence, I was forced to let go this hypothesis.

But it was not my intention to attempt to account for, but only to state facts in this article. One thing I had forgotten in the proper place to mention, namely, that all were foreigners (Hollanders) by birth, but had been in this country some seven or eight years, during which, they had resided in the place where these facts occurred, or in that vicinity.

To me, these were novel and interesting cases, but if they shall be so to others of the profession, I do not know. I have not attempted to do more in my statement of them, than briefly to describe those symptoms which were unusual. W.

